# Economic and Psychosocial Impact of COVID-19 in the Hispanic Community Health Study/Study of Latinos

**DOI:** 10.1089/heq.2022.0211

**Published:** 2023-03-27

**Authors:** Carmen R. Isasi, Linda C. Gallo, Jianwen Cai, Marc D. Gellman, Wenyi Xie, Gerardo Heiss, Robert C. Kaplan, Gregory A. Talavera, Martha L. Daviglus, Amber Pirzada, Sylvia Wassertheil-Smoller, Maria M. Llabre, Marston E. Youngblood, Neil Schneiderman, Eliseo J Pérez-Stable, Anna M. Napoles, Krista M. Perreira

**Affiliations:** ^1^ Department of Epidemiology and Population Health, Albert Einstein College of Medicine, Bronx, New York, USA.; ^2^ Department of Psychology, San Diego State University, San Diego, California, USA.; ^3^ Department of Biostatistics, University of North Carolina at Chapel Hill, Chapel Hill, North Carolina, USA.; ^4^ Department of Psychology, University of Miami, Coral Gables, Florida, USA.; ^5^ Department of Epidemiology, University of North Carolina at Chapel Hill, Chapel Hill, North Carolina, USA.; ^6^ Institute for Minority Health Research, University of Illinois Chicago, Chicago, Illinois, USA.; ^7^ National Institute on Minority Health and Health Disparities and Division of Intramural Research, National Heart, Lung and Blood Institute, Bethesda, Maryland, USA.; ^8^ Division of Intramural Research, National Institute on Minority Health and Health Disparities, National Institutes of Health, Bethesda, Maryland, USA.; ^9^ Department of Social Medicine, UNC School of Medicine, Chapel Hill, North Carolina, USA.

**Keywords:** COVID-19, Hispanic/Latino, economic hardship, mental health

## Abstract

**Objectives:**

To examine the prevalence and correlates of economic hardship and psychosocial distress experienced during the initial phase of the coronavirus disease 2019 (COVID-19) pandemic in a large cohort of Hispanic/Latino adults.

**Methods:**

The Hispanic Community Health Study/Study of Latinos (HCHS/SOL), an ongoing multicenter study of Hispanic/Latino adults, collected information about COVID-19 illness and psychosocial and economic distress that occurred during the pandemic (*N*=11,283). We estimated the prevalence of these experiences during the initial phase of the pandemic (May 2020 to May 2021) and examined the prepandemic factors associated with pandemic-related economic hardship and emotional distress using multivariable log linear models with binomial distributions to estimate prevalence ratios.

**Results:**

Almost half of the households reported job losses and a third reported economic hardship during the first year of the pandemic. Pandemic-related household job losses and economic hardship were more pronounced among noncitizens who are likely to be undocumented. Pandemic-related economic hardship and psychosocial distress varied by age group and sex. Contrary to the economic hardship findings, noncitizens were less likely to report pandemic-related psychosocial distress. Prepandemic social resources were inversely related to psychosocial distress.

**Conclusions:**

The study findings underscore the economic vulnerability that the pandemic has brought to ethnic minoritized and immigrant populations in the United States, in particular noncitizens. The study also highlights the need to incorporate documentation status as a social determinant of health. Characterizing the initial economic and mental health impact of the pandemic is important for understanding the pandemic consequences on future health. Clinical Trial Registration Number: NCT02060344

## Introduction

In the United States, the pandemic of coronavirus disease 2019 (COVID-19), caused by severe acute respiratory syndrome coronavirus-2 (SARS-COV-2) infection, has been characterized by striking racial and ethnic disparities in overall prevalence, disease severity, and mortality.^1,2^

The impact of COVID-19 has been especially striking in Black/African American, Hispanic/Latino, and American Indian/Alaskan Native populations.^1,2^ Adverse social determinants of health have driven inequities in the burden and outcomes of COVID-19 among these populations,^3^ by shaping the ability to practice physical distancing and to obtain access to timely testing, vaccination, and high-quality health care. The economic consequences of the pandemic also appear to be driven by these structural inequities.^1,4^ Hispanic/Latino families have faced disproportionate job losses, as the service and retail industries were decimated, and/or were more likely to work in essential jobs characterized by heightened exposure risks and no guaranteed sick time or time off for testing and vaccination.^5–7^

Although economic improvements have been noted in the United States, Hispanic/Latino individuals and other underserved groups continue to experience disproportionate food insufficiency, an inability to cover rent, and trouble meeting household expenses.^8^

In addition, the pandemic and efforts to contain it appear to have fostered a new mental health crisis.^9,10^ A Centers for Disease Control and Prevention (CDC) survey in June 2020 found that more than 4 in 10 adults had at least 1 mental or behavioral health concern, with 31% reporting symptoms of anxiety or depression.^11^ These concerns occurred more often among young adults, racial/ethnic minorities, and those with preexisting mental health concerns.^12,13^

Additional research is needed to understand the consequences of the pandemic on Hispanic/Latino families to guide targeted prevention and intervention programs and predict future physical and mental health consequences of COVID-19 and future pandemics. The current study attempted to address this gap in knowledge using data from the Hispanic Community Health Study/Study of Latinos (HCHS/SOL). HCHS/SOL represents a unique opportunity to study the initial impact of the COVID-19 pandemic on the Hispanic/Latino population given the well-characterized cohort from different areas of the U.S. and representing multiple heritage groups. Characterizing the initial experience is important, as the first year of the pandemic was the most disruptive socially and economically. Furthermore, the cohort has been followed since 2008–2011, allowing examination of prepandemic influences on economic and psychosocial distress.

The goals of the study were to examine economic hardship, emotional distress (depression and anxiety symptoms), and social isolation during the COVID-19 pandemic, and to explore prepandemic social determinants of health, including U.S. citizenship status as key social determinant of health in this large and predominantly immigrant cohort.

## Methods

HCHS/SOL is an ongoing population-based cohort study of 16,415 Hispanic/Latino adults who were selected using a multistage probability sampling design from four U.S. metropolitan areas (Chicago, IL; Miami, FL; Bronx, NY; San Diego, CA) with representation of the major Hispanic/Latino subpopulations (i.e., Mexican, Puerto Rican, Cuban, Dominican, Central or South American). Details about the aims and methodology of HCHS/SOL are published elsewhere.^14,15^ In brief, at the baseline in-person examination (Visit 1; 2008–2011), participants completed a comprehensive assessment of cardiometabolic risk or protective factors, questionnaires in their language of preference, and a blood draw for DNA extraction and other biomarkers. A second in-person examination occurred ∼6 years later (Visit 2; 2014–2017). A third examination is ongoing.

The HCHS/SOL team conducted a 15-min phone survey to capture COVID-19 economic and psychosocial consequences. The survey was completed by 11,283 individuals (72.5% response rate) between May 2020 and May 2021; 59% of surveys were completed between May and August 2020, 30% between September and December of 2020, 9% between January and March 2021, and 2% during April and May 2021. The study was conducted with Institutional Review Board approval from each of the institutions involved in the study.

## Measures

### COVID-19 experiences and consequences

#### COVID-19 pandemic-related job loss and economic hardship

Individuals were asked to report whether they or someone in the household lost their job at any point since the start of the COVID-19 pandemic in the United States (March 2020). If so, they were categorized as experiencing a household job loss. Specific scores for financial (difficulty paying for utilities, phone, or internet, range=0–3), housing (range=0–2), and food insecurity (range=0–1) were also calculated. The primary economic hardship variable was a composite score of report of difficulties paying for basic needs (housing, food, phone or internet, utilities). A numeric score ranging from 0 to 6 was assigned based on the number of hardships reported. For analysis, hardship scores were dichotomized as 0 versus ≥1.

#### COVID-19 pandemic-related psychosocial distress

Anxiety and depression symptoms were assessed using the validated 2-item Patient Health Questionnaire (PHQ-2) screeners for depression^16,17^ and Generalized Anxiety Disorders 2-item (GAD-2).^18^ Respondents indicate how often they experienced cardinal depression and anxiety symptoms on a scale of 0 (not at all) to 3 (nearly every day). PHQ-2 and GAD-2 scale scores of ≥3 (range=0–6) were used to indicate elevated depression or anxiety symptoms.^17,18^ Individuals with scores ≥3 on either or both scales were categorized as having elevated psychological distress. Social isolation was evaluated using a single item assessing how often the respondent felt isolated from others in the past 2 weeks, using the same response format as the PHQ-2 and GAD-2 (range=0–3). A scale score of ≥2 (felt isolated most or all days) was used to indicate elevated social isolation.

### Prepandemic factors

#### Prepandemic psychological distress

At HCHS/SOL Visit 2, depression symptoms were assessed with the Centers for Epidemiological Studies in Depression-10 item scale,^19^ which has been shown to be valid and reliable in the HCHS/SOL.^20^ Anxiety symptoms were assessed with the GAD-7, a reliable and valid screener for probable clinically significant anxiety in the general population.^21,22^ Recommended cut scores of ≥10 were used to indicate prior moderate/severe symptoms of depression or anxiety.^19,22^ Individuals were classified as having prepandemic elevated psychological distress if their Visit 2 CESD-10 and/or GAD-7 scores were ≥10.

#### Prepandemic social resources

At HCHS/SOL Visit 2, participants completed the Interpersonal Support Evaluation List-12 item (ISEL-12),^23^ a measure of available functional (i.e., perceived) social support, which has been shown to be reliable and valid in the HCHS/SOL cohort. The upper quartile was used to define individuals with “high” social resources before the start of the COVID-19 pandemic.

#### Citizenship

During the HCHS/SOL Visit 2, participants reporting that they were not U.S. citizens were asked whether they were legal permanent residents, had applied for legal permanent residency, held another type of visa, or if none of these situations applied. Consistent with prior methods applied in HCHS/SOL,^24,25^ citizen status was classified as: U.S.-born citizen (i.e., all individuals born in the 50 U.S. states/DC or U.S. territories); naturalized citizen; documented noncitizen (i.e., holding or applying for legal permanent residency or another visa); or likely undocumented noncitizen (not holding or applying for a visa).

Sociodemographic factors, including sex, Hispanic/Latino background, and place of birth were collected at baseline. Duration of U.S. residence and age were calculated at the time of COVID-19 interview. Annual household income and educational attainment data were obtained from HCHS/SOL Visit 2.

### Data analyses

Unadjusted prevalence of self-reported economic hardship, and psychosocial distress were summarized using complex survey procedures to account for the HCHS/SOL design. Multivariable log linear models with binomial distributions were used to estimate the prevalence ratio (PR) to examine the prepandemic factors associated with each of the outcomes of interest. These models included the following covariates: age groups at the time of COVID-19 interview, sex, field center, Hispanic/Latino background, income, education attainment, citizenship, preexisting psychosocial distress, and preexisting social resources. The entire HCHS/SOL cohort at baseline, excluding those who died during the follow-up period (*N*=851), was included. Missing covariates and outcomes were handled using multiple imputation.^26^ The imputation model included all covariates in the log linear model and variables potentially related to missingness.

These auxiliary variables included: language preference, marital status, health insurance status, cardiovascular disease, hypertension, diabetes, physical activity, smoking, alcohol intake, mental health status, physical health status, social acculturation, anxiety and depression symptoms, time between baseline visit and COVID-19 interview, and baseline exam sampling weight. The fully conditional specification method was used for imputation.^27^ Logistic or linear regression methods were used depending on whether variables were categorical or continuous. Each variable with missingness was imputed sequentially until all covariates were complete.

This imputation process was repeated 10 times resulting in 10 full complete datasets.^26^ Ninety-five percent confidence intervals (95% CIs) for the regression coefficients were constructed based on the combined estimates and the associated standard errors. PRs and the associated 95% CIs were then calculated by exponentiating the combined estimates of the regression coefficients and their associated 95% CIs. All the analyses were performed using SAS 9.4 software (SAS Institute, Cary, NC) and SUDAAN software Release 11 (RTI International, Research Triangle Park, NC).

## Results

### Study population

The characteristics of the COVID-19 interview sample and the entire HCHS/SOL cohort are presented in Table 1. In brief, the COVID-19 interview sample included 7143 females and 4140 males between the ages of 27 and 88. The majority of the sample was born outside of the United States (states/DC/U.S. territories); about 30% were naturalized citizens, 29% were documented noncitizens, and 17% were likely undocumented noncitizens. Participants were predominantly of lower socioeconomic status; 39% reported an annual household income <$20,000 before the pandemic and about 36% did not graduate from high school. The distributions of characteristics in the COVID-19 interview sample was similar to those for the entire HCHS/SOL cohort at baseline.

**Table 1.** Unweighted Sociodemographic Characteristics of the Coronavirus Disease 2019 Interview Sample and the Entire Hispanic Community Health Study/Study of Latinos Cohort

		COVID-19 interview sample (***N***=11,283)	HCHS/SOL cohort (***N***=16,415)
	*N* (COVID-19 interview sample)	% (SE)	% (SE)
Sex			
Female	7143	63.3 (0.42)	59.9 (0.37)
Male	4140	36.7 (0.42)	40.1 (0.37)
Age group			
<35	911	8.3 (0.29)	
35–44	1293	11.8 (0.37)	
45–64	5550	50.6 (0.55)	
65+	3210	29.3 (0.53)	
Field center			
Bronx	2476	21.9 (1.08)	25.1 (1.15)
Chicago	2944	26.1 (1.40)	25.2 (1.29)
Miami	2998	26.6 (2.06)	24.8 (1.95)
San Diego	2865	25.4 (1.38)	24.9 (1.28)
Hispanic/Latino Background			
Dominican	959	8.5 (0.54)	9.0 (0.54)
Central American	1213	10.8 (0.71)	10.6 (0.63)
Cuban	1710	15.2 (1.44)	14.3 (1.34)
Mexican	4598	40.8 (1.49)	39.4 (1.38)
Puerto Rican	1633	14.5 (0.67)	16.6 (0.69)
South American	826	7.3 (0.41)	6.5 (0.34)
Mixed/other/missing	344	3.0 (0.17)	3.6 (0.16)
Household income			
<$20,000	3436	39.1 (0.75)	40.3 (0.69)
$20,000–$50,000	3887	44.2 (0.59)	43.3 (0.53)
>$50,000	1473	16.7 (0.63)	16.4 (0.59)
Education			
<High school	3128	35.6 (0.73)	36.3 (0.68)
High school graduate	2058	23.5 (0.50)	23.5 (0.45)
Some college	1897	21.6 (0.51)	21.4 (0.47)
College degree	1692	19.3 (0.61)	18.9 (0.55)
Citizenship			
U.S.-born citizen	2815	24.9 (0.83)	28.4 (0.82)
Naturalized citizen	3362	29.8 (0.57)	23.5 (0.46)
Noncitizen—documented	3237	28.7 (0.73)	29.1 (0.73)
Noncitizen—likely undocumented	1869	16.6 (0.65)	19.0 (0.61)
Duration of U.S. residency			
Born in U.S.	2690	24.5 (0.83)	35.0 (0.98)
25 or more years	4375	39.8 (0.65)	34.3 (0.56)
<25 years	3920	35.7 (1.06)	30.7 (1.02)

COVID-19, coronavirus disease 2019; HCHS/SOL, Hispanic Community Health Study/Study of Latinos; SE, standard error.

### COVID-19 pandemic-related economic hardship

The prevalence of household job loss and economic hardship during the COVID-19 pandemic overall and by sociodemographic factors are depicted in Table 2. As predicted, individuals in HCHS/SOL were deeply impacted by the economic consequences of the pandemic. A high percentage (46%) reported household job loss and elevated economic hardship (35%). Among the types of economic hardship, 19% reported financial insecurity, 19% reported housing insecurity, and 23% reported food insecurity. The oldest age group (≥65 years) reported less household job loss and economic hardship than younger groups. The highest economic hardship was reported among noncitizens who are likely undocumented (54%). This group also reported the highest percentage of household job losses, financial, housing, and food insecurity.

**Table 2.** Unadjusted Prevalence^a^ of Coronavirus Disease 2019-Related Socioeconomic Hardships by Key Sociodemographic Characteristics

	Self/household job loss (***N***=10,964)	Economic hardship (***N***=10,853)	Financial insecurity (***N***=11,283)	Housing insecurity (***N***=11,283)	Food insecurity (***N***=10,926)
	% (SE)	% (SE)	% (SE)	% (SE)	% (SE)
Overall	45.8 (0.84)	35.0 (0.91)	19.1 (0.69)	19.1 (0.68)	22.8 (0.74)
Sex					
Female	46.1 (1.00)	37.1 (0.95)	20.6 (0.78)	19.7 (0.75)	24.2 (0.80)
Male	45.5 (1.20)	32.2 (1.22)	17.3 (0.89)	18.2 (0.95)	21.1 (1.01)
Age group (age at covid interview)					
<35	51.6 (2.28)	32.9 (2.02)	20.6 (1.77)	18.8 (1.56)	20.0 (1.70)
35–44	57.0 (1.71)	41.0 (1.99)	24.1 (1.62)	27.0 (1.74)	24.8 (1.59)
45–64	51.1 (1.05)	38.6 (1.21)	21.8 (0.98)	21.4 (0.96)	26.3 (1.03)
65+	21.4 (1.12)	23.4 (1.25)	10.6 (0.78)	9.5 (0.77)	16.0 (1.06)
Field center					
Bronx	35.8 (1.67)	38.7 (1.75)	24.2 (1.57)	22.7 (1.59)	26.3 (1.56)
Chicago	53.0 (1.38)	40.9 (1.56)	23.9 (1.25)	21.2 (1.20)	27.4 (1.21)
Miami	50.4 (1.39)	37.7 (1.43)	17.2 (0.97)	21.0 (1.01)	25.3 (1.19)
San Diego	46.2 (1.70)	24.9 (1.63)	13.7 (1.32)	11.9 (1.16)	14.1 (1.19)
Hispanic/Latino Background					
Dominican	41.9 (2.66)	38.5 (2.47)	24.0 (2.05)	24.5 (2.20)	23.8 (1.96)
Central American	58.2 (2.38)	47.2 (2.20)	23.0 (2.13)	25.3 (1.90)	36.1 (1.96)
Cuban	45.5 (1.74)	33.0 (1.78)	14.4 (1.09)	17.9 (1.23)	22.1 (1.39)
Mexican	50.7 (1.32)	33.1 (1.52)	19.7 (1.31)	17.5 (1.18)	20.9 (1.30)
Puerto Rican	23.7 (1.59)	31.1 (1.83)	19.2 (1.52)	15.6 (1.30)	21.8 (1.56)
South American	58.6 (2.32)	44.9 (2.72)	21.8 (2.16)	24.4 (2.16)	27.8 (2.22)
Mixed/other/missing	47.1 (3.77)	31.5 (3.82)	17.0 (2.52)	20.5 (3.31)	14.9 (2.28)
Household income^*^					
<$20,000	37.9 (1.41)	40.2 (1.33)	22.7 (1.11)	21.6 (1.11)	27.3 (1.20)
$20,000–$50,000	51.8 (1.36)	37.0 (1.41)	19.7 (1.05)	19.4 (1.11)	23.2 (1.03)
>$50,000	45.1 (1.80)	18.4 (1.44)	9.9 (1.13)	11.0 (1.13)	10.3 (1.15)
Education^*^					
<High school	40.5 (1.66)	39.9 (1.55)	22.3 (1.38)	20.7 (1.41)	27.8 (1.53)
High school graduate	50.7 (1.68)	39.5 (1.73)	20.9 (1.47)	21.5 (1.44)	25.0 (1.36)
Some college	47.8 (1.78)	31.3 (1.81)	16.2 (1.24)	16.2 (1.28)	20.1 (1.40)
College degree	43.0 (1.73)	25.7 (1.65)	14.5 (1.23)	14.5 (1.14)	15.9 (1.46)
Citizenship					
U.S.-born citizen	34.9 (1.51)	27.6 (1.29)	16.4 (1.03)	14.4 (0.95)	17.8 (1.04)
Naturalized citizen	41.4 (1.42)	29.6 (1.51)	15.1 (1.03)	14.3 (0.95)	18.4 (1.20)
Noncitizen—documented	50.5 (1.39)	37.0 (1.37)	18.7 (0.98)	20.8 (1.10)	24.7 (1.12)
Noncitizen—likely undocumented	63.7 (1.79)	52.8 (1.88)	31.1 (1.86)	31.6 (1.82)	35.5 (2.00)
Duration of U.S. residency					
Born in U.S.	34.7 (1.53)	27.8 (1.32)	16.5 (1.06)	14.6 (0.97)	18.0 (1.09)
25 or more years	44.2 (1.34)	33.5 (1.33)	18.6 (1.13)	17.7 (1.15)	21.1 (1.15)
<25 years	54.5 (1.15)	40.9 (1.38)	22.2 (1.08)	24.0 (1.12)	27.5 (1.12)

^a^ All prevalences were calculated using complex survey procedures and accounted for sampling weights, clustering, and stratification.

As displayed in Table 3, multivariable analysis showed that the likelihood of experiencing economic distress varied by age, sex, field center, and citizenship status. Specifically, there was a graded association of age with the oldest age group (65+ years old) least likely and the youngest age group (<35 years old) most likely to experience a household job loss [PR_<35 years_=2.35 (2.06, 2.68)] and economic hardship [PR_<35 years_=1.76 (1.56, 1.98)] with 65+ as the reference group. The likely undocumented noncitizens were the most vulnerable to job losses [PR=1.39 (1.22, 1.58)] and economic hardship [PR=1.53 (1.30, 1.81) with U.S.-born citizens as the reference group. Individuals with the lowest prepandemic household incomes were more likely than those with higher incomes to report economic hardship [PR_<20,000/year_=1.72 (1.49, 1.99)] with >$50,000/year as the reference group. However, job losses did not vary by prepandemic household income.

**Table 3.** Prevalence Ratios^a^ for Prepandemic Factors Associated with Pandemic-Related Economic and Psychosocial Distress

	Household job loss	Economic hardship	Psychosocial distress^*^	Social isolation
	PR (95% CI)	PR (95% CI)	PR (95% CI)	PR (95% CI)
Sex				
Female	1.06 (1.00–1.13)	1.09 (1.01–1.17)	1.29 (1.14–1.46)	1.23 (1.04–1.46)
Male	Ref.	Ref.	Ref.	Ref.
Age group (age at covid interview)				
<35	2.35 (2.06–2.68)	1.76 (1.56–1.98)	0.82 (0.65–1.03)	1.02 (0.77–1.34)
35–44	2.27 (2.02–2.55)	1.75 (1.54–1.98)	0.95 (0.77–1.17)	1.18 (0.94–1.50)
45–64	2.02 (1.81–2.25)	1.60 (1.46–1.76)	1.16 (1.04–1.30)	1.21 (1.03–1.42)
65+	Ref.	Ref.	Ref.	Ref.
Field center				
Bronx	0.99 (0.87–1.13)	1.45 (1.21–1.73)	1.31 (1.00–1.73)	1.04 (0.73–1.48)
Chicago	1.14 (1.05–1.23)	1.43 (1.23–1.66)	1.16 (0.94–1.44)	0.98 (0.75–1.26)
Miami	1.28 (1.12–1.47)	1.58 (1.32–1.89)	1.34 (1.04–1.73)	1.04 (0.70–1.54)
San Diego	Ref.	Ref.	Ref.	Ref.
Hispanic/Latino background				
Dominican	0.88 (0.75–1.03)	0.96 (0.79–1.17)	1.22 (0.93–1.60)	1.21 (0.81–1.81)
Central American	0.99 (0.87–1.12)	1.00 (0.87–1.16)	1.08 (0.83–1.40)	1.19 (0.82–1.71)
Cuban	0.82 (0.71–0.96)	0.81 (0.68–0.96)	1.30 (1.03–1.65)	1.54 (1.05–2.27)
Mexican	Ref.	Ref.	Ref.	Ref.
Puerto Rican	0.66 (0.54–0.80)	1.02 (0.83–1.26)	1.10 (0.83–1.45)	1.43 (1.01–2.03)
South American	1.05 (0.93–1.19)	1.07 (0.92–1.23)	1.10 (0.84–1.43)	1.14 (0.78–1.67)
Mixed/other/missing	0.94 (0.77–1.14)	0.97 (0.76–1.23)	1.04 (0.70–1.54)	0.76 (0.44–1.30)
Household income				
<$20,000	0.92 (0.81–1.03)	1.72 (1.49–1.99)	1.18 (0.95–1.47)	1.18 (0.92–1.52)
$20,000–$50,000	1.06 (0.96–1.17)	1.61 (1.38–1.89)	0.98 (0.81–1.19)	1.05 (0.79–1.40)
>$50,000	Ref.	Ref.	Ref.	Ref.
Educational attainment				
<High school	1.00 (0.90–1.10)	1.24 (1.08–1.43)	0.93 (0.78–1.11)	0.96 (0.76–1.21)
High school graduate	1.10 (1.00–1.21)	1.22 (1.07–1.40)	0.97 (0.78–1.20)	1.01 (0.79–1.31)
Some college	1.09 (1.00–1.19)	1.13 (0.97–1.33)	0.97 (0.78–1.20)	1.07 (0.84–1.36)
College degree or greater	Ref.	Ref.	Ref	Ref.
Citizenship				
U.S.-born citizen	Ref.	Ref.	Ref	Ref.
Naturalized citizen	1.14 (0.99–1.32)	1.24 (1.05–1.46)	0.72 (0.58–0.89)	0.93 (0.73–1.19)
Noncitizen—documented	1.23 (1.08–1.40)	1.35 (1.17–1.57)	0.75 (0.60–0.95)	0.89 (0.69–1.14)
Noncitizen—likely undocumented	1.39 (1.22–1.58)	1.53 (1.30–1.81)	0.67 (0.51–0.89)	0.81 (0.61–1.07)
Prepandemic psychological distress	0.99 (0.92–1.07)	1.21 (1.12–1.31)	1.91 (1.66–2.20)	1.96 (1.63–2.35)
Prepandemic social resources	1.02 (0.98–1.05)	0.96 (0.93–1.00)	0.91 (0.86–0.97)	0.88 (0.80–0.96)

^*^ Psychosocial distress was operationalized as having either elevated depression symptoms, anxiety symptoms, or both.

^a^ PRs were calculated based on log linear models with binomial distributions and accounted for sampling weight, clustering, and stratification. Models simultaneously adjust for all variables in the table. Multiple imputation was used to address missing data.

CI, confidence interval; PR, prevalence ratio.

### COVID-19 pandemic-related psychosocial distress

As shown in Table 4, elevated depression and anxiety symptoms varied by sex and age group, with women and older groups most affected. About 14% of the total population reported feelings of social isolation, with a higher prevalence of elevated social isolation among women and lower prevalence among those <35 years old. There were also differences across field centers, with those in the Bronx and Miami appearing to experience more psychosocial distress relative to those in Chicago and San Diego. Higher prevalence of psychosocial distress and social isolation were observed among the lowest household income group. While U.S.-born citizens showed the least economic hardship; they showed the highest prevalence of elevated anxiety and depression symptoms, and social isolation, relative to noncitizens.

**Table 4.** Unadjusted Prevalence^a^ of Elevated Psychosocial Distress Experienced by Key Sociodemographic Characteristics

	Elevated anxiety symptoms (***N***=10,933)	Elevated depression symptoms (***N***=10,928)	Social isolation (***N***=10,925)
	% (SE)	% (SE)	% (SE)
Overall	16.0 (0.60)	10.3 (0.49)	13.7 (0.59)
Sex			
Female	18.9 (0.77)	12.6 (0.69)	15.5 (0.73)
Male	12.2 (0.79)	7.3 (0.56)	11.5 (0.83)
Age group			
<35	11.2 (1.29)	5.2 (0.77)	10.8 (1.34)
35–44	13.9 (1.25)	7.6 (0.91)	12.8 (1.34)
45–64	18.5 (0.98)	11.8 (0.79)	15.1 (0.91)
65+	16.0 (1.02)	12.9 (0.99)	13.8 (1.05)
Field center			
Bronx	22.5 (1.45)	15.0 (1.37)	17.3 (1.38)
Chicago	12.1 (0.81)	7.8 (0.65)	11.3 (0.86)
Miami	17.4 (0.98)	11.8 (0.78)	15.1 (0.92)
San Diego	10.3 (0.96)	5.3 (0.55)	10.2 (1.20)
Hispanic/Latino background			
Dominican	19.7 (2.24)	13.3 (1.92)	14.7 (2.04)
Central American	14.1 (1.47)	8.6 (1.18)	12.5 (1.19)
Cuban	18.8 (1.26)	13.0 (0.94)	16.6 (1.24)
Mexican	10.8 (0.82)	5.9 (0.50)	10.1 (0.94)
Puerto Rican	24.4 (1.85)	18.2 (1.90)	21.7 (1.96)
South American	15.7 (1.99)	8.6 (1.66)	12.3 (1.64)
Mixed/other/missing	16.6 (2.61)	8.2 (1.80)	7.9 (1.83)
Household income^*^			
<$20,000	20.9 (1.19)	17.0 (1.09)	18.7 (1.14)
$20,000–$50,000	14.0 (0.85)	8.0 (0.71)	12.8 (0.90)
>$50,000	12.6 (1.22)	6.3 (0.80)	10.4 (1.24)
Education^*^			
<High school	16.6 (1.09)	13.0 (1.05)	14.9 (1.04)
High school graduate	16.2 (1.32)	11.3 (1.19)	15.0 (1.45)
Some college	16.7 (1.47)	11.2 (1.20)	14.5 (1.52)
College degree	16.1 (1.30)	8.6 (0.90)	13.1 (1.25)
Citizenship			
U.S.-born citizen	20.2 (1.25)	13.4 (1.11)	16.8 (1.21)
Naturalized citizen	15.1 (1.06)	10.2 (0.95)	14.0 (1.16)
Noncitizen—documented	15.0 (0.94)	9.2 (0.71)	12.8 (0.91)
Noncitizen—likely undocumented	11.8 (1.16)	6.9 (0.92)	9.8 (1.01)
Duration of U.S. residency			
Born in U.S.	20.6 (1.28)	13.6 (1.13)	16.8 (1.24)
25 or more years	15.2 (0.91)	10.3 (0.82)	13.4 (0.89)
<25 years	13.5 (0.83)	7.9 (0.58)	12.1 (0.83)

^a^ All rates were calculated using complex survey procedures and accounted for sampling weight, clustering, and stratification.

In multivariable analyses (Table 3), women [PR=1.29 (1.14, 1.46)] and those with elevated prepandemic depression symptoms [PR=1.91 (1.66, 2.20)] were more likely to have elevated psychological distress during the COVID-19 pandemic. However, higher prepandemic social resources were inversely associated with psychosocial distress [PR=0.91 (0.86, 0.97)]. Similar associations were observed for prepandemic predictors of COVID-19 pandemic experiences of social isolation.

## Discussion

This study demonstrated high levels of COVID19-related economic hardship in the longest ongoing cohort of Hispanic/Latino adults, with almost half of the households reporting job losses and a third reporting economic hardship in the first year of the pandemic. Important variations were noted by citizenship, which highlights documentation status as a key social determinant of health.^28–30^ Specifically, pandemic-related household job losses and economic hardship were more pronounced among noncitizens who are likely to be undocumented. These findings underscore the economic vulnerability that the pandemic has brought to immigrant populations in the United States, and in particular noncitizens. These findings are not entirely surprising given that many federal and state economic relief measures excluded undocumented individuals.^5,31^ Even among documented immigrants, the fear of losing documented status or a path to citizenship may prevent Hispanic/Latino adults from accessing the financial aid for which they are eligible.

Furthermore, the study revealed that individuals with lower household incomes, who are likely to have been financially insecure before the pandemic, were the most likely to experience economic hardship during the pandemic. Of all the factors that define economic hardship, food insecurity was the most prevalent, affecting noncitizens the most. Our results are in line with previous reports. The Urban Institute reported that greater economic losses and material hardship following the COVID-19 pandemic occurred among households with a noncitizen in the family.^32^ Another national survey also found that those with the lowest prepandemic incomes were more likely to experience economic hardship during the initial pandemic period.^33^ Taken together, these findings are worrisome indicators of how the U.S. is failing to protect the most vulnerable in our society.

Younger adults were more likely than adults ≥65 years of age to report household job losses and economic hardship. This finding is not surprising since most individuals over 65 are retired, but it may have important implications for the future. In addition, nonskilled and service work are common occupations among HCHS/SOL individuals, occupations that are less conducive for remote work and may have offered less job security during the initial phases of the pandemic. On the other hand, young adulthood represents a period when individuals start families and have their most productive years. Facing economic adversity as a young adult may have important long-term socioeconomic and health consequences later in life. Ultimately, the long-term health and socioeconomic consequences may represent another means through which the COVID-19 pandemic has magnified health disparities experienced by Hispanic/Latino communities and could significantly diminish life expectancy advantages previously seen among the overall Hispanic/Latino population.

While the economic impact was more apparent in the younger adults and noncitizens, these groups were less likely to report psychosocial distress or social isolation. In part, the difference for noncitizens may reflect the lower prepandemic depression and anxiety symptom levels reported previously in this group,^25,34,35^ given that prepandemic psychosocial distress was a robust predictor of pandemic-related elevated psychosocial distress and isolation in the current study.

Notably, the rates of elevated pandemic-related depression and anxiety symptoms in the current study were lower than those reported in a national survey that used similar brief screening instruments;^36^ however, this national survey did not report differences in symptoms by racial and ethnic groups. In HCHS/SOL, prepandemic resilience factors (social support, family cohesion) protected against psychosocial distress and social isolation during the COVID-19 pandemic. Fostering social resources could be an important approach to mitigate the adverse mental health effects of natural disasters, like the COVID-19 pandemic, in the future.

The study has limitations that are important for interpreting the findings. To minimize participant burden during a very stressful period, assessments used brief instruments that do not capture all nuanced features of social, economic, and mental health consequences experienced during the pandemic, which may underestimate effect sizes. Although a strength of the study is the availability of economic and psychosocial factors before the pandemic, these assessments were obtained over 7 years ago. The use of different survey instruments in prior assessments also limited the ability to directly measure changes as a result of the COVID-19 pandemic. In addition, the study captures the effects of the first year of the pandemic and does not shed light on the impact of subsequent waves and variants with a much larger rate of infections, the emergence of long-COVID, and differences in economic reopening.

Despite these limitations, the study underscores the need to account for the profound societal effects of the pandemic in the analysis of ongoing cohorts, especially for surveillance of underserved and vulnerable populations.

Trajectories of cardiovascular disease and other health conditions may be adversely affected by the disruptions caused by COVID-19 to individuals and societies. Follow-up for health events continues in the HCHS/SOL, and future studies will capture the long-term health effects of the pandemic and examine how the adverse socioeconomic consequences will shape future disease risks and outcomes in this and other cohorts.^37^

### Health equity implications

The pandemic brought to light profound inequalities in the United States and the lasting impact of cumulative disadvantages, such as limited financial and social resources that are exacerbated under stress.^38,39^ There is recent discussion about including documentation status in assessments of social determinants of health.^28–30^ This study supports this by showing that noncitizens were most vulnerable to economic hardships. Looking into the future, it is important to remember that health disparities do not emerge suddenly—they are a manifestation of systemic factors operating across many decades, made worse by any emergent or novel disease or natural disaster. In the case of COVID-19, the forces leading to health and economic inequities served to widen existing health disparities among racial and ethnic minority and immigrant populations,^40,41^ who shouldered the worst of the pandemic. The path to health equity will not be achieved unless we start addressing their root causes with preventive measures and policies that address longstanding structural inequities.

## Abbreviations Used

CDC: Centers for Disease Control and Prevention
COVID-19: coronavirus disease 2019
GAD-2: generalized anxiety disorders 2-item
HCHS/SOL: Hispanic Community Health Study/Study of Latinos
ISEL-12: Interpersonal Support Evaluation List-12 item
PHQ-2: two-item Patient Health Questionnaire
PR: prevalence ratio
SARS-COV-2: severe acute respiratory syndrome-coronavirus-2
SE: standard error
